# Histological age estimation of the eggs of *Calliphora vicina* Robineau Desvoidy (Diptera: Calliphoridae)

**DOI:** 10.1080/20961790.2017.1404707

**Published:** 2017-12-26

**Authors:** Michael Pais, Melanie S. Archer

**Affiliations:** Department of Forensic Medicine, Victorian Institute of Forensic Medicine, Monash University, Southbank, Victoria, Australia

**Keywords:** Forensic science, forensic entomology, Calliphoridae, *Calliphora vicina*, eggs, histology, embryology

## Abstract

Aging blow fly eggs can be critical to a forensic investigation, but there are currently no forensically useful timelines describing internal anatomical changes in embryological development. This is partly due to the lack of an economical, rapid and technically simple histological technique to allow mass production of slides for research and casework. We present a histological method that uses a slightly modified standard laboratory processing run with 1 h fixation in 10% formalin, 2 h softening in Molliflex and Haemotoxylin and Eosin (H&E) staining. We also present a summary of the internal anatomical changes that can be visualized using our technique in the developing eggs of *Calliphora vicina* Robineau-Desvoidy (Diptera: Calliphoridae). We examined eggs from at least three different females grown at 15 °C and sampled at 6 h intervals, and eggs grown at 20 °C and sampled at 3 h intervals. Blind aging trials demonstrated that it is possible to accurately age material grown at 20 °C to within 6 h (but attempts to further narrow this interval resulted in errors in one-third of cases). It was also possible to see sufficient anatomical detail to age eggs preserved for forensic casework 5, 8, 9, 10 and 11 years previously. Additionally, we determined that section quality was improved by 5 s fixation in hot water prior to preservation in ethanol. However, hot water fixation for longer than this increased the level of section artefact.

## Introduction

Blow fly species (Diptera: Calliphoridae) are usually the first to colonize a dead body, and many forensically important species are oviparous [1]. This means that the ability to estimate post oviposition age of blow fly eggs can provide crucial information for investigators in medico-legal cases [2–4]. This is especially true within the first few hours or days after initial body colonization, when blow fly eggs may be the only entomological evidence present.

The blow fly life cycle can be divided into four main stages: egg, larva, pupa and finally the adult. The larval stage can be further sub-divided into the first, second and third instar. The durations of these stages are all temperature dependent and will be faster in general if the temperature is warmer. The forensically important species of blow fly, *Calliphora vicina* Robineau-Desvoidy (Diptera: Calliphoridae) spends approximately 5%–6% of its total juvenile life in the egg stage [5].

There have been several taxonomic studies on the morphology of blow fly egg chorions, both using scanning electron microscopy [6–8] and light microscopy [9]. External changes have also been documented in several species for the purpose of aging the developing embryo [10–12]. But there is still a paucity of information on how to age blow fly eggs using internal anatomy, although the internal changes potentially allow finer resolution of aging than external changes, especially if the two methods are used together. This is because there are more changes, and they occur more rapidly than external changes [12].

It has been found that aging blow fly eggs to within 2 h with gene expression is possible, and the *cs*, *bcd* and *sll* genes have been found to be suitable [13]. However, optimal results with this technique require preservation in a special fixative, such as RNA Later™, and the technique is also expensive.

Standard histology is an easy and practical method for examining internal egg development. However, ease of use is ultimately dependent on the experience of the operator and the facilities available, so is optimal when performed by a qualified histologist working in a histology laboratory. Standard histology comprises paraffin embedding and Haematoxylin and Eosin (H&E) staining. This service is widely available throughout forensic and anatomical pathology laboratories, and is very economical. It would also be ideal if slides could be prepared using eggs collected into ethanol, as this is currently the standard in forensic entomology [14], and also if the technique worked on “cold case” material that had been preserved for prolonged periods. Finally, it would be advantageous if eggs could be processed for embedding on the standard tissue run, alongside other material routinely being processed in diagnostic or autopsy pathology work.

Routine histological processing would not only allow rapid sectioning of eggs for casework, but would also facilitate research to produce forensically useful timeline data. Large numbers of eggs could be sectioned simultaneously, and a variety of planes of section and features could be captured on the same slide, and within each egg batch.

There has been very limited work on the internal morphological sequence of embryogenesis in forensically important calliphorids. Early work on *C. vicina* histology was performed by Lowne [15,16]. It should be noted that *C. erythrocephala* is a junior synonym of *C. vicina* and some literature refers to this species name. Work on *Lucilia sericata* (Meigen) (Diptera: Calliphoridae) embryology was carried out by Fish [11,17–19] and Davis [20], and on *Phormia regina* (Meigen) (Diptera: Calliphoridae) by Auten [21]. Some of the most detailed work was performed by Van Der Starre-Van Der Molen on *C. vicina* [12,22,23], and these papers include comprehensive stage descriptions, along with a very approximate developmental timeline at 22 °C. However, only 16–80 embryos were studied per developmental stage in the major work by this author [12], most likely due to the labour intensive nature of the methods used. Much of the work on *Drosophila melanogaster* Meigen (Diptera: Drosophilidae) also provides useful parallels with the processes and structures seen in calliphorid development [24–27]. In particular, the Bownes’ stages [28,29] provide a useful division of changes, albeit describing a species in a different dipteran family [10].

The terminology of Van Der Starre-Van Der Molen [12] will be used in the following description. Both Van Der Starre-Van Der Molen and Bownes made very comprehensive stage descriptions although the stages did not correspond exactly (Table 1). The normal sequence of development begins with a yolk filled and fertilized egg at time 0, which is usually the time of oviposition, but may occur beforehand in the case of precocious egg development [30]. Divisions of nuclei derived from the anteriorly located zygote nucleus begin after time 0. These nuclei, destined to become the first embryonic cells, lack outer membranes. They are located within the yolk mass, and are surrounded by a halo of plasm, which is devoid of yolk granules (“plasm island”). At first, the nuclei multiply via repeated divisions, and they spread throughout the yolk. However, most soon begin to migrate to the periphery of the egg (migration stage) to form a syncytial outer layer (blastoderm stage). Further nuclear divisions occur, and the pole cells (future primordial germ cells) are formed. Cell membranes then form around the naked nuclei. Work with *Drosophila* suggests that the materials for the cell membranes are derived from vesicles arrayed in the periplasm (outer plasm layer) of the egg [27].

**Table 1.** Comparison of the Van der Starre-Van der Molen stages of blowfly development in *Calliphora vicina* [12] and the Campos-Ortega and Hartenstein version of Bownes’ stages of development in *Drosophila melanogaster* [28,29]. Comparison is approximate, as the stages do not correspond exactly.

Van der Starre-Van der Molen stages	Bownes stages (version by Campos-Ortega and Hartenstein)
Cleavage	Stages 1–2
Migration	Stages 2–4
Blastoderm	Stage 5
Pseudo-segmentation	Stages 6–8
True Segmentation	Stages 9–10
Beginning abdominal segmentation	Stages 11–12
Segmentation head piece	Stages 13–14
Beginning midgut	Stages 14–15
Thick midgut	Stage 15
Thin midgut	Stage 16
Trachea mouth hooks	Stage 17

The next major stage is “pseudosegmentation”, which is characterized by considerable cell migration during gastrulation. This eventually results in the formation of the three germ layers (endoderm, ectoderm and mesoderm). The germ band, which is a ventral amalgamation of germ layers, extends along an anterior to posterior gradient during this stage. The cephalic furrow, which demarcates the head segment, also forms and organ rudiments begin to differentiate. The pole cells move dorsally, and then begin to descend into the embryo along with the invaginating mid-dorsal “proctodeum” (future hind gut and anus). The “true segmentation” stage follows, and is marked by the initial formation of the head segments. There is accompanying progression and virtual completion of organ rudiment formation, and these include the precursor structures for the neural, gastrointestinal and tracheal systems. The stomodeum (future foregut and mouth), which will become the mouth, also begins to invaginate [12,23].

“Beginning abdominal segmentation” proceeds next, and results in the formation of the thoracic segments, followed by the abdominal segments. The anus precursor (proctodeum entrance) also moves caudally, and the stomodeum continues to invaginate. The “segmentation head piece” stage follows, and is characterized by continuing abdominal segmentation, invagination and ongoing differentiation of the head structures, and the appearance of a prominent hind gut loop. Importantly, the anterior and posterior parts of the gut fuse, and midgut formation proceeds. The head segments invaginate into the thorax, in keeping with the “acephalic” appearance of the calliphorid maggot. Further modification of the gut occurs during the “beginning midgut” stage, along with dorsal closure of the embryo. There is epithelial fusion of the edges of the defect caused by the eventual retraction of the germ band [26]. Further elongation and looping of the gut occurs during the “thick midgut” stage, followed by the “thin midgut” stage. The final stage is the “trachea mouth hooks” stage, which lasts until hatching. During this time, the organs mature in preparation for hatching, and the chitinized structures become pigmented. At the point of egg eclosion, histological sections would reveal a pharate maggot.

Many of the early histological studies demonstrated subtle developmental features in exquisite detail, and were the first to elucidate the complex changes described above. However, the methods used were time-consuming, and involved some chemicals that are particularly toxic and potentially dangerous, such as Carnoy's solution and picric acid [12,22,23]. Also, multiple specialized staining techniques were used, and these were tailored to specific embryological stages in some cases [12,22]. This would not be as suitable for forensic work where the reference material is of unknown age prior to analysis. However, different stains could potentially be applied to serial sections following assessment of an initial H&E section.

As mentioned above, relatively small numbers of eggs were sectioned for some foundation studies describing blow fly embryology [12]. Others examined larger total numbers of eggs, but it is unclear exactly how many eggs were examined at each stage [18,21]. Additionally, the illustrations for early papers may consist of photographs or idealized black and white schematic illustrations that best display the features of interest [11,12,17–23]. These can be difficult to relate to material that is less than perfect, or that does not show all of the anatomical features that allow aging. In addition, the temperatures at which much of the early work was carried out, and the timing of anatomical development, were not precisely recorded, thus rendering these studies unsuitable for forensic use. Finally, some of the most detailed studies pre-date the introduction of the terminology used today and were still seeking to understand basic processes that have become better characterized in the modern era [15,16,31,32]. Their findings are therefore sometimes difficult to interpret and compare across studies.

Van Der Starre-Van Der Molen noted that the eggs of *C. vicina* develop synchronously at early stages, but diverge later [12]. It was therefore recommended that a sequence of stages, rather than a timeline, was the most suitable way to conceptualize embryogenesis. However, this is unsuitable for forensic purposes, and a timeline produced from large numbers of eggs would be expected to provide a statistically robust measure of developmental variation during embryogenesis. The degree to which precocious egg development occurs could also be further studied in this fashion.

A histological method is presented here for sectioning and staining *C. vicina* eggs. This is part of a large scale study aimed at documenting the timing of embryogenesis in this species, along with other calliphorids. The decision to study *C. vicina* is based on two important factors. First, *C*. *vicina* can be an early colonizer of cadavers, both outdoors and indoors [1], and can therefore provide an accurate estimate of minimum post-mortem interval. Second, *C. vicina* is a forensically important cosmopolitan species [1] that is cool weather active and present in abundance in Melbourne.

This is a proof of concept study that shows histology is a viable method for determining the age of blow fly eggs especially those of *C. vicina*, and while a developmental timeline at two temperatures is presented, this is not yet detailed. The aim is to devise a histological method that can be integrated with standard forensic histology laboratory slide preparation protocols, and that would not require labour intensive preparatory steps. Additionally, the method should be applicable to eggs sampled straight into ethanol, which is the current standard forensic practice [14]. However, we also explored whether hot water fixation produces superior sections, and whether samples that have been in storage for up to 11 years can also be examined using this technique. We applied our method to eggs grown at both 15 °C and 20 °C in order to demonstrate that sufficient anatomy can be seen throughout development to allow the eventual construction of a precise timeline in future work.

## Methods

### Culture methods

Adult *C. vicina* (F12) were obtained from the Forensic Entomology Research Laboratory of the University of Wollongong, New South Wales (NSW). The original colony was established from flies captured in Silverdale, NSW (33°56'32''S 150°34'48''E). Adults were kept at the Victorian Institute of Forensic Medicine (VIFM) at 20 °C constant temperature, in three cages that were each 15 cm wide, 25 cm high and 30 cm long. Cages had 23 cm × 15 cm panels cut out of the sides and curtain material affixed, as well as curtain material sleeves for access to cages. Sugar and water were provided *ad libitum*, and kangaroo mince (VIP Pet Mince) was also given as a protein source for ovarian maturation. Colonies were egged at least once per week into 70 mL round containers filled with 20 g fresh kangaroo mince topped by cotton wool.

### Embryology at 15 °C and 20 °C

Eggs were grown at 15 °C and 20 °C to determine whether key anatomical features could be seen at both temperatures throughout development. Three containers of mince and eggs, each taken from one of three different colony cages, were transferred into an Axyos IM1000 RG (Queensland, Australia) temperature controlled cabinet within 15 min of laying, and were maintained at either 20 °C or 15 °C (with a temperature variation of ±0.5 °C) in complete darkness, depending on the experimental regime. At each collection time, a 00 size paint brush was used to transfer up to approximately 200 eggs from each container (depending on batch size) to an Eppendorf tube filled with 80% ethanol. Great care was taken to minimize the risk of contamination between samples by cleaning the brush thoroughly in running water after each transfer, and inspecting it for entrapped eggs. Only one sample per container was taken, and each replicate was ended following sampling. Therefore, for each time point, three separate egg batch samples (replicates) were collected, each one obtained from a different colony cage to ensure that the progeny of at least three different females was obtained.

The sampling interval for the 20 °C eggs was 3 h (to within 15 min of age) until hatching, commencing with freshly laid (T_0_). Additional collections of approximately 200 eggs each were also made at 20 °C at the one (T_1_) and two (T_2_) hour stages because this is described as a period of considerable morphological change in the existing literature [12,28]. The same collecting process was followed for *C. vicina* eggs grown at 15 °C, and these were collected at 9 h intervals, with one additional collection at 38.5 h in order to assess the changes during this period of rapid late development which had been ascertained from the literature [12]. The T_0_ stage collection was shared between the 15 °C and 20 °C experiments since these were freshly oviposited eggs.

### Histological method

Prior to processing eggs from the 15 °C and 20 °C time series, a histological method was derived by trial and error. Large numbers of *C. vicina* eggs were grown at room temperature (19 °C–20 °C), and collected for pilot trials into Eppendorf tubes filled with 80% ethanol at early (0–6 h), middle (7–20 h) and late (20–24 h) stages of development. The final methodology described below was devised with trial and error variation of chemical fixatives and softening agents. The quality of sections was compared qualitatively by an observer blinded to the technique being used. Both the degree of artefact produced, and the clarity of anatomy seen were taken into account. Once established, the following methodology was applied to all experiments.

Ethanol preserved eggs were transferred for processing from Eppendorf tubes by using a 00 size paint brush into a histology cassette, and placed between two biopsy pads. The brush was washed between transfers. Cassettes were then placed in 10% neutral buffered formalin for 1 h and processed on either a Tissue-Tek 5 or Tissue-Tek 6 (Sukura Finetek, USA) processing machine overnight (Table 2). It was also apparent from initial trials that specimen shrinkage, and other sectioning artefacts (e.g. shatter) were minimized by exposure to 10% formalin for 1 h prior to processing, as opposed to using no formalin, using overnight formalin or two days of formalin.

**Table 2.** Histological processing schedule for *Calliphora vicina* eggs (run overnight in a Tissue Tek 5 or 6 machine). The pressure/vacuum was on for every station.

Station	Process	Time (h)	Temperature (°C)
1	Formalin	1	40
2	70% ethanol	2	40
3	100% ethanol	1	40
4	100% ethanol	1	40
5	100% ethanol	1	40
6	100% ethanol	2	40
7	100% ethanol	2	40
8	Xylene	2	40
9	Xylene	2	40
10	Xylene	2	40
11	Wax	1	60
12	Wax	1	60
13	Wax	1	60
14	Wax	1	60

The cassettes were removed from the processing machine the following morning, and eggs were embedded in paraffin wax at 60 °C, using a Tissue-Tek TEC embedding centre (Sukura Finetek, USA). The eggs were embedded as they fell in a random orientation, with between 15 and >50 eggs from one of three females embedded in a single block (dependent on batch size) for each time point. One block per replicate of each experimental treatment was made so actual egg numbers varied between a minimum of 15 eggs and a maximum of >150 eggs for each time point. Blocks were carefully trimmed to the most superficial aspect of the face on a Reichert-Jung 2030 microtome (Leica Biosystems Nussloch GmbH, Germany) using S35 Feather Microtome Blades (Feather Safety Razor, Japan). The block surfaces were then immersed in Mollifex (VWR International Ltd., England) for 2 h to allow for surface softening of the embedded eggs. The volume of softening agent used was only enough to cover the trimmed block face. Molliflex was superior to Nair (Church and Dwight, Australia) and Decal (7% Nitric Acid made up in distilled water) in pilot experiments because it softened the chorion, thereby minimising shattering, without appearing to affect the egg contents. Decal in particular caused shrinkage of the embryological tissues.

Levels were cut to 4 μm thick using a Zeiss Hyrax M40 microtome (Carl Zeiss Oberkochen, Germany). The levels were 48 μm apart and the block was cut right through. Sections were cut slowly because this greatly minimized shatter artefact, which was due to the chorion (this still presented a challenge to cut even after softening in Molliflex). Sections were individually floated onto a warm water bath at 45 °C and picked up on Menzel-Glasser Superfrost White glass microscope slides (Gerhard Menzel GmbH, Germany), which were allowed to rest on a hot-plate at 50 °C before being transferred to staining racks. The full slide racks were left overnight in a 60 °C Thermoline Scientific Laboratory Incubator (Thermoline Scientific, Australia) to allow total adherence of the section to the glass slide. They were then transferred to the Leica ST 5020 staining machine (Leica Microsystems Pty Ltd., Germany) for staining with H&E [33]. Coverslips were applied using the Leica CV 5030 coverslipping machine (Leica Microsystems Pty Ltd., Germany). Menzel-Glasser 24 mm × 50 mm coverslips were mounted onto the slides using Leica CV Mounting Medium.

### Blind aging trial

Following the establishment of the histological method, and the 15 °C and 20 °C timeline, preliminary validation of the work was sought by aging six samples of unknown age in a blind trial. Samples of approximately 200 eggs each were produced from the Silverdale colony at University of Wollongong, and were sent to VIFM with codes to identify them. The samples were between T_0_ and T_24_, and had been preserved directly in 80% ethanol. The authors separately estimated the age in hours for each sample, and allocated them to developmental stages. They then compared their answers, and reached a consensus where there were discrepancies. The ages of the blind samples were then obtained from Associate Professor James Wallman (University of Wollongong) by matching the sample codes.

### Hot water fixation

Hot water fixation prior to preservation in ethanol has already been employed in the preservation of eggs for examination of external embryo changes [10]. We conducted trials to determine whether it improves the histological section quality of blow fly eggs. First, eggs were reared at 20 °C in the temperature controlled cabinet for 8 and 12 h, respectively. Three egg samples of approximately 200 eggs each were fixed for 10 s at each time interval in freshly boiled water. These eggs were then placed into 80% ethanol. Three duplicate samples were placed directly in 80% ethanol at each collection time. One section per sample with levels to tissue extinction was produced using the standard method described above, and the amount of artefact, and the anatomical detail visible was assessed and compared qualitatively. Assessment was based on crispness of cellular detail, and freedom from tissue loss and artefact.

A second trial was performed in order to ascertain whether the length of hot water fixation time is an important factor in section quality. Two *C. vicina* colonies were provided with an egging dish, and checked 6 h later. Three samples were taken from each of the colonies of approximately 200 eggs each; one sample was hot water fixed for 5 s, one for 20 s and finally one for 90 s, before being placed into 80% ethanol. One block per replicate was produced with multiple levels to extinction, and these were compared qualitatively to assess anatomical detail and artefact.

### Aged casework material

We assessed whether our standard histological technique is suitable for use on casework samples that had been stored for variable periods. Five egg samples were sourced from prior forensic entomology cases for which the evidence had passed its mandated minimum storage time, and for which all legal proceedings had concluded. Cases were 5, 8, 9, 10 and 11 years old. Species was determined where possible from the original forensic entomology case file. All samples were preserved in 80% ethanol, and the 5-year-old case had been fixed in hot water for an unspecified time prior to preservation in ethanol.

## Results

### Embryology at 15 °C and 20 °C

**Figure 1.**
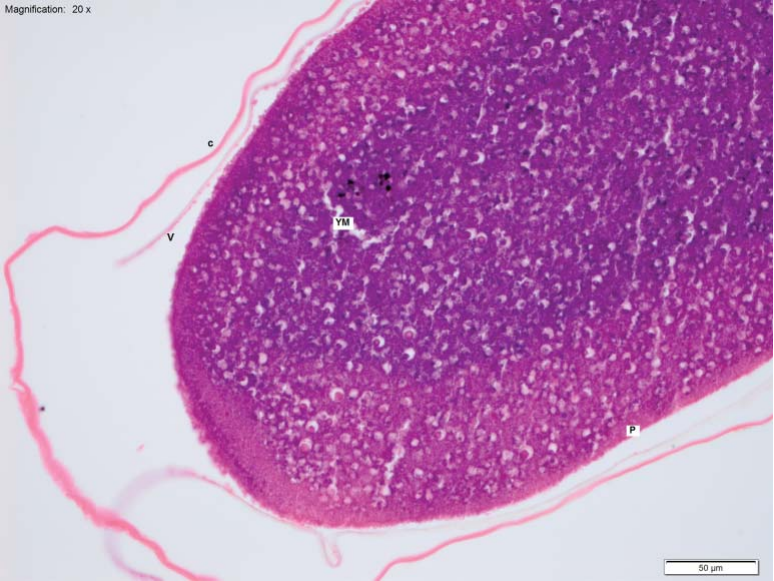
Longitudinal section of *Calliphora vicina* egg at T_0_ (immediately post oviposition), “Cleavage” stage (H&E × 200). The egg mostly comprises an amorphous, homogenous yolk mass (YM). A thin periplasm layer (P) surrounds the YM, and the egg is enclosed in the inner vitelline membrane (V), and outer chorion (C). This egg was fixed in 80% ethanol. Temperature at collection was 20 °C.

**Figure 2.**
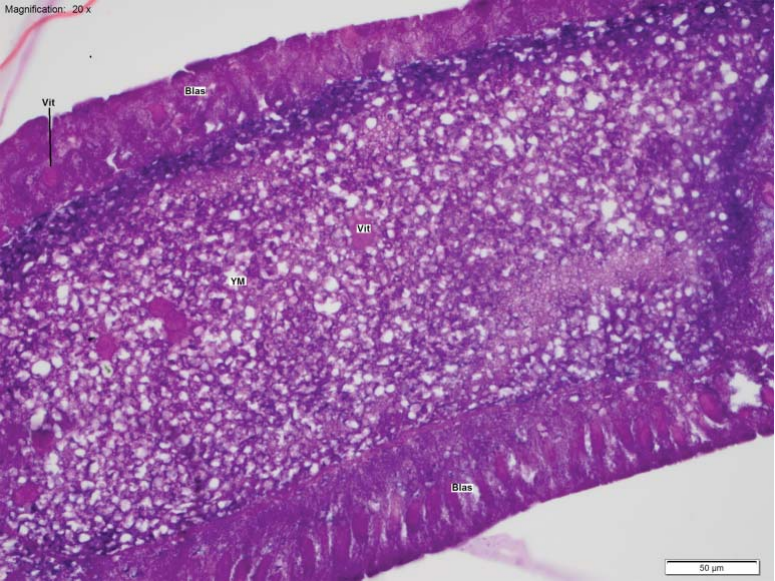
Longitudinal section of *Calliphora vicina* egg at T_3_ (3 h post oviposition), “Blastoderm” stage (H&E × 200). The blastoderm (Blas) lines the perimeter of the egg. The yolk mass (YM) is centralized. Vitellophages (Vit) are also clearly visible. This egg was fixed and stored in 80% ethanol. Temperature at collection was 20 °C.

**Figure 3.**
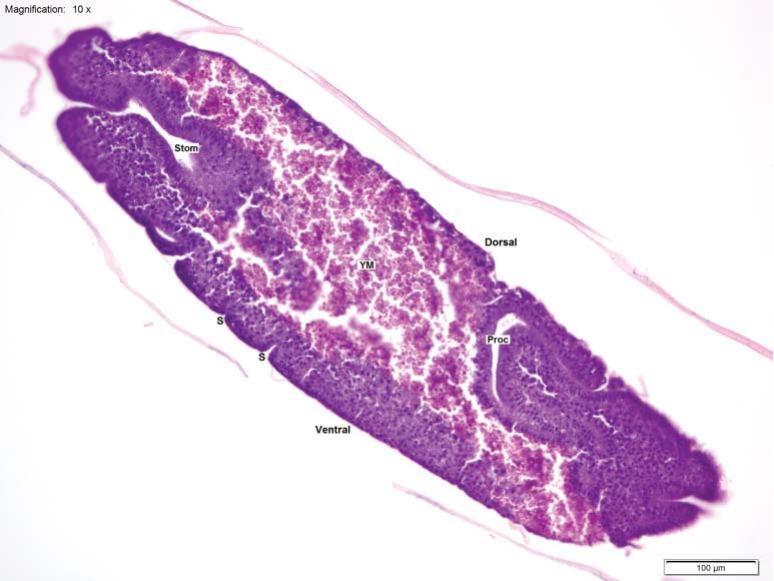
Longitudinal section of a *Calliphora vicina* egg at T_12_ (12 h post oviposition), “Segmentation Head Piece” stage (H&E × 100). The proctodeum (Proc) and stomadeum (Stom) are clearly visible. The yolk mass (YM) has moved ventrally, and the beginnings of both the thoracic and abdominal segments (S) are visible. This egg was hot water fixed and stored in 80% ethanol. Note the superior cellular definition compared with Figures 1–2 and 4–6. Temperature at collection was 20 °C.

**Figure 4.**
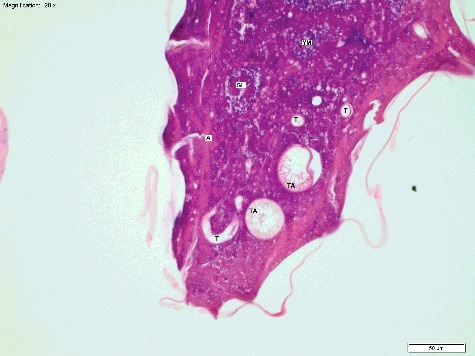
Longitudinal section of a *Calliphora vicina* egg at T_21_ (21 h post oviposition), “Thin Midgut” stage (H&E × 200). The tracheoles (T) and taenidia (TA) have formed. Gut loops (GL) are reducing in diameter, and the gut is coiling, with the remnants of yolk mass (YM) within the lumen. Apodemes (A) are also visible, with attached muscle bands. This egg was fixed in 80% ethanol. Temperature at collection was 20 °C.

**Figure 5.**
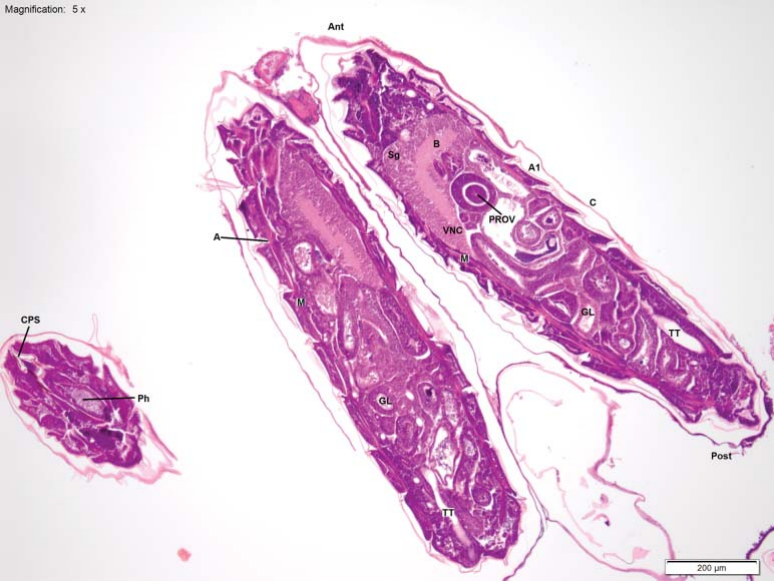
Longitudinal section of a *Calliphora vicina* egg at T_24_ (24 h post oviposition) in the “Trachea Mouth Hooks” stage, the embryo is almost ready to eclose (H&E × 50). The brain (B) and ventral nerve cord (VNC) have condensed so that the distal end lies within segment A1. The gut loops (GL) are clearly defined and thin. The sub-oesophageal ganglion (Sg) has developed and the tracheal trunks (TT) are clearly visible. Apodemes (A), muscle (M) and the proventriculus (Prov) are well defined. An adjacent egg has been sectioned transversely, showing the cephalopharyngeal skeleton (CPS) and the pharynx (Ph). Surrounding each egg is the chorion (C). This egg was fixed in 80% ethanol. Temperature at collection was 20 °C.

We used the developmental stages and anatomical features defined by Van Der Starre-Van Der Molen [12,22,23] to compare with our own slides. First, we extracted a summary of the main features and processes described in this work, along with the approximate timing of development at 22 °C. This included identification of structures that were transient, and those that were ongoing throughout embryonic development (Table 3). We then compared the structures identified by us using our simple technique with those seen by Van Der Starre-Van Der Molen using optimized techniques (Table 4 and Figures 1–5). We determined that we could see the majority of structures, although there were some features we did not encounter. We did not see the zygote nucleus, oosome or polar granules (cleavage stage), and we did not see the Malphigian tubes (true segmentation stage), or the gonads (segmentation headpiece stage). There were also some features that were seen infrequently, and these were the pole cells, cephalic and oblique furrows, salivary glands and gastric caeca. Finally, there was a subset of features that were not seen by us until their development was advanced. The anterior and posterior midgut remnants could not be distinguished from the surrounding cells until the segmentation headpiece stage, and even then, they were seen only rarely, and only with hot water fixation. The earliest developmental stages of the muscle, tracheoles, proventriculus, gastric caeca and neural tissue were also not seen (Tables 3 and 4).

**Table 3.** Main anatomical features and developmental stages identified by Van der Starre-Van der Molen [12,22,23], and estimated stage timing (h) at 22 °C. Features that are ongoing for the duration of development, and those that are transient over one to two stages are separated. Ongoing features are listed only once when they appear, whereas transient features are listed at each stage they are seen.

	Main anatomical features			
Stage	Ongoing	Transient	Developmental processes and explanation of anatomy	Time (h)
Cleavage	Yolk mass, chorion, vitelline membrane.	Periplasm layer, plasm islands, zygote nucleus.	Yolk mass comprises yolk granules and yolk plasm. Periplasm layer surrounds the yolk mass (cytoplasm with no yolk granules). The entire egg is surrounded by the vitelline membrane, which is encapsulated by the chorion (outermost layer). Early nuclear division occurs within plasm islands which do not contain yolk granules, but have a cleavage nucleus centrally.	0–1.75
Migration	Primary vitellophages.	Cleavage nuclei, plasm islands.	Cleavage nuclei and their plasm islands migrate to egg periphery and form syncytial blastoderm by the end of the stage. Increase in plasm islands and continued nuclear cleavage occurs, and primary vitellophages are established (nuclei that remain in the central yolk mass instead of migrating to the periphery).	1.75–2.25
Blastoderm	Cell membranes, secondary vitellophages, nucleoli.	Pole cells.	Cell membranes form, and nucleoli become visible. Secondary vitellophages appear (nuclei that are pushed from periphery back into yolk mass when cell membranes form). Pole cells form and bulge posteriorly by the end of the stage.	2.25–3.75
Pseudo-segmentation	–	Gastrulation groove/germ band, polar concavity, anterior and posterior midgut rudiments, proctodeum, Cephalic furrow (and 3-4 temporary oblique furrows).	Gastrulation groove forms, rapidly becomes tubular, and then solid as the lumen disappears (forms mesoderm). Mesoderm and overlying ectoderm are together known as the “germ band”. Polar concavity forms (columnar cells) and rapidly becomes the posterior midgut rudiment (PMR). Proctodeum forms (invagination that later forms the hind gut and anus), along with the anterior midgut rudiment (AMR).	3.75–5
True segmentation	Malphigian tubules, major body segments, rudiments of brain, tracheal system, and muscle.	Stomodeum, proctodeum, cephalic furrow.	Cephalic furrow deepens. Malphigian tubes, and tracheal rudiments form. Muscle begins to develop, and the entrance to the gastrointestinal tract (stomodeum) begins to invaginate. Neuroblasts become visible in head segments (beginning of brain and ventral nerve cord).	5–6.5
Beginning abdominal segmentation	Full body segmentation, salivary glands, crop, hind gut dorsal loop.	Stomodeum, proctodeum.	Formation of the abdominal and thoracic segments occurs (3 × thoracic, 8 × abdominal segments). Proctodaeum moves posteriorly, and hind gut loops dorsally. Yolk mass pushed upwards, and mainly into first abdominal segments as germ band shortens. Tracheal pits (entrances to the tracheal system) invaginate. Rudimentary salivary glands and crop form.	6.5–8.5
Segmentation – head piece	Oesophagus, pharynx, gonads, tracheoles.	–	Head involutes into thorax, and brain moves posteriorly into thoracic segments, while yolk mass shifts into middle abdominal segments. Formation of midgut completes by fusion of the AMR and PMR. Respiratory system continues to differentiate, and gonads begin to form.	8.5–12
Beginning midgut	Proventriculus, gastric caeca, imaginal leg discs, ventral nerve cord.	–	Dorsal closure of embryo. Hindgut elongates. Oesophagus bulges into gut lumen to form early proventriculus. Four gastric caeca develop at the base of proventriculus. Two main tracheal trunks begin forming. Ventral nerve cord appears.	12–13
Thick midgut	Muscle, tracheal trunks, brain, sub-oesophageal ganglion with central nervous system.		Hindgut forms multiple smaller loops. Muscle fibres (myofibrils) develop.	13–15
Thin midgut	Taenidia.	–	Gut coiling continues. Cuticle thickens further. Taenidia develop in trachea. Condensation of the ventral nerve cord proceeds. Myofibrils continue to develop.	15–18
Trachea mouth hooks	–	–	Last vitellophage nuclei digested, along with remaining yolk mass in midgut. Myofibrils thicken. Pigmented chitinous structures develop. Cuticle thickens rapidly. Tracheae fill with air. Ventral nerve cord finally condenses to lie between T_3_ and A1.	18–21

-:no data

**Table 4.** Anatomical features and developmental stages identified using routine processing with Haematoxylin and Eosin staining. The range of times (h) at which each stage and feature was seen is given for development at 15 °C and 20 °C. Features that were seen rarely (<5 eggs), or at only one temperature, are indicated.

Stage	Time at 15 °C (h)	Time at 20 °C (h)	Anatomical features	Developmental process
Cleavage	0–9	0–2	Periplasm, cleavage nuclei, yolk mass, vitelline membrane, plasm islands, chorion.	Yolk mass and periplasm only at T_0_, with gradual increase of plasm islands and cleavage nuclei.
Migration	9	1–2	Primary vitellophages, cleavage nuclei, plasm islands.	Nuclear migration to egg periphery.
Blastoderm	9	3	Cell membrane formation, secondary vitellophages, pole cells (seen rarely), nucleoli.	Blastoderm formation (with and without cell membranes).
Pseudo-segmentation	9	3^a^, 6	Cephalic and oblique furrows (seen rarely), proctodeum, gastrulation groove in transverse sections only, polar concavity (seen rarely).	–
True segmentation	9	3^a^,6–9^b^	Proctodeum, stomodeum.	–
Beginning abdominal segmentation	18	6–9	Proctodeum, tracheal pits (seen rarely at 20 °C only), hind gut loops, rudimentary abdominal segments.	–
Segmentation head piece	18	12, 21^b^	Tracheoles, anterior and posterior midgut rudiments meeting (seen rarely), complete midgut.	Yolk mass movement into middle abdominal segments.
Beginning midgut	18	12–15	Proventriculus, rudimentary muscle development (20 °C only), and apodemes.	–
Thick midgut	18–27	15–18, 24^b^	Muscle well developed, apodemes, pharynx, gastric caeca, hind gut loops, tracheal trunks.	–
Thin midgut	27–38.5	15^a^, 18–21, 24^b^	Spine bands (unpigmented), brain and suboesophageal ganglion, long ventral nerve cord, multiple narrow calibre gut loops, tracheal taenidia.	–
Trachea Mouth Hooks	18^a^, 36–45	18^a^, 21–24	Spine bands and mouth hooks with pigmented chitin, fully condensed ventral nerve cord.	–

^a^Features and stages that most likely represent precocious egg development are indicated with.

^b^Features and stages that are thought to have represented examples of delayed development or death of the embryo are indicated with.

-:no data

The timing of the stages at 20 °C was roughly comparable with the timing seen at 22 °C by Van Der Starre-Van Der Molen [12], although comparison and fine resolution of stage timing was not an aim of this study. The 15 °C egg series showed a lower level of resolution in the timing of stage change due to the low sampling frequency, however the same sequence of development, and same structures were seen. The stages also occupied roughly the same proportion of development at 15 °C and 20 °C. This means that development can be at least assigned to broad increments that comprise ranges of around 6 h at 20 °C or roughly 9 h at 15 °C. Examples of eggs that showed markedly accelerated development were occasionally seen at both temperatures, and these are hypothesised to be precocious (<5% of the total). By contrast, eggs were occasionally seen in the 20 °C series that were underdeveloped compared with their cohort (<5% of the total). These may represent dead or diseased eggs (Table 4).

The general quality of sections produced with this processing method was of a reasonable standard, but did not approach the crisp cellular detail seen by other authors using more labour intensive techniques [11,12,17–19,22,34]. However, the major aim of this study was to locate sufficient anatomical detail in order to estimate egg age. We therefore had very positive overall results, with tissue loss preventing the aging of embryos in only 7% of slides, and insufficient anatomy preventing age estimation in a further 2% of slides (at 20 °C). Each replicate block had multiple levels (slides), therefore all blocks could be aged using at least one level.

Histological artefacts were also present in some sections with variable degrees of severity. We encountered tissue shrinkage (tissue section size is smaller than size of the tissue in the block), shatter (caused by the microtome blade vibrating as the tissue block passes across), tissue loss (where not all of the tissue on the face of the block is transferred to the section) and knife lines (caused by using a damaged microtome blade to cut the section, or the tissue containing a hard component that does not section). Often the chorion had been pulled away from the embryo, which is the unfortunate result of simultaneously attempting to cut a hard surface, such as the chorion, and a soft surface, such as the embryo. Finally, numerous “floaters” were seen in sections (tissue that does not belong to specimen being cut, and that may be a block or water bath contaminant). Some were recognizably human tissue, most often brain, or were skeletal muscle and bacteria that probably originated from the egging medium.

The human tissue appeared because our eggs were cut using microtomes and floated out on water baths that were previously used to cut sections of human tissue. In routine forensic work, this meant that some specks of human tissue left in the water bath were picked up on the slide with the fly egg sections. This was rare as the water in the bath was changed every morning because floaters are not permitted in routine Coronial sections as part of strict quality control. Also, we were confident that there were no egg contaminants in our sections because the egg samples were cut sporadically, often with significant time-frames between cutting batches. However if large batches were to be sectioned, consideration should be given to changing the water bath. We would also change the water bath in future before cutting egg sections after the routine Coronial work has been done.

### Blind aging trial

All of the broad age range estimations (6 h increments) were correct for the blind egg aging trial. However, we had discrepancies of 3 h for two of the narrow age range estimates (Table 5). One was an early stage sample (T_6_), and the other was a late stage sample (T_24_). Narrow age ranges usually spanned 3 h, but age was given correctly to the hour in one case (only because the answer was T_0_, and there were no precocious specimens; Table 5). We were not always able to see the features required to help narrow down the broad age estimates with confidence (e.g. a mid-sagittal longitudinal section of the ventral nerve cord). However, we were able to assign a developmental stage in all cases.

**Table 5.** Comparison of actual egg ages with estimated ages (h) in blind identification trial. A broad age range in 6 h increments is provided, along with an age range as narrow as we were able to assign. The developmental stages seen, as described by Van der Starre-Van der Molen [12] are also listed.

Sample	Actual age (h)	Estimated broad age range (h)	Estimated narrow age range (h)	Estimated stage(s)
A1	6	0–6	1–3	Cleavage, Migration, blastoderm
A2	24	18–24	18–21	Thin midgut
A3	3	0–6	0–3	Cleavage, migration and early blastoderm
A4	14	12–18	12–15	Beginning midgut, thick midgut
A5	0	0-6	0	Cleavage
A6	18	18–24	18–21	Thin midgut

### Hot water fixation

Qualitative assessment of hot water fixation revealed that 5 s in hot water prior to ethanol preservation produced optimal section quality (Figure 3). Longer times in hot water resulted in increased tissue shrinkage, presumably due to heat coagulation. Tissues and organs became unrecognizable after 90 s, and results became inferior to ethanol after only 20 s in hot water. Fixation for 10 s gave better results than ethanol alone, but was inferior to 5 s fixation.

### Aged casework material

**Figure 6.**
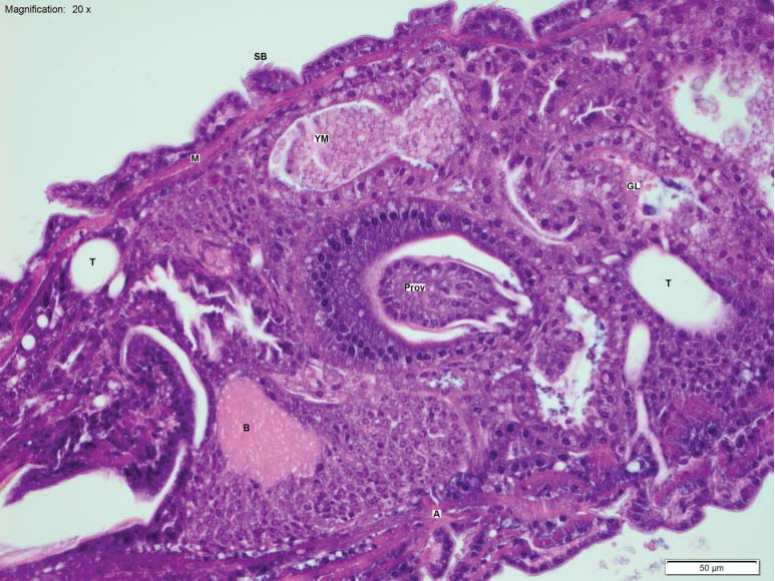
Longitudinal section of a *Chrysomya varipes* egg from a forensic case (fixed and stored in 80% ethanol for 11 years, H&E × 200). Temperature at collection unknown. The section shows “Trachea Mouth Hooks” stage, with clearly visible proventriculus (Prov), muscle (M), tracheole (T), neural tissue (B), apodeme (A) and non-pigmented spine bands (SB).

The processing technique developed in this study also worked well with stored eggs between 5 and 11 years old. The 5-year-old case had been hot water fixed for an unknown length of time, and the tissue was markedly coagulated. This case was therefore the most difficult to analyse. The remaining cases showed good anatomical and cellular detail (Figure 6); specimen storage time was not predictive of section quality. Stages represented were “beginning of abdominal segmentation”, “beginning midgut”, “thin midgut” and “trachea mouthhooks”. Features seen were yolk mass, proctodaeum, muscle, trachea, neural tissue, mouthhooks and spine bands. Chitin pigmentation was also preserved at the “trachea mouth hooks” stage in one case sample that had been stored for 8 years (Table 6).

**Table 6.** Stored evidential material (eggs) kept for 5, 8, 9, 10 and 11 years. Estimated developmental temperature (°C) is provided, where available, either as a mean or range. The post-mortem interval (days) is provided where it is known, and the entomological minimum post-mortem interval estimate (PMI_min_) is provided instead where the actual post mortem interval is unknown.

Storage (years)	Month of death	Temperature (°C)	Habitat	Cause of death	Post-mortem interval (days)	Species reared from eggs	Embryo anatomy seen	Stage(s) present
5	Aug	14–20	Urban (house)	Effects of fire	<4	*Calliphora stygia*	Muscle, tracheae and yolk mass. Coagulated tissue	Thin midgut (at least)
8	Oct	16	Woodland	Blunt force trauma to head	4	None	Tracheoles, taenidia, tracheal trunks, pigmented chitin (cephalopharyngeal skeleton, spine bands), muscle, residual yolk mass in gut loops, neural tissue, pharynx, mature proventriculus	Trachea mouth hooks
9	Sep	10	Urban (shed)	Effects of fire	3	*Calliphora stygia*	Proctodaeum, early proventriculus	Beginning abdominal segmentation, beginning midgut
10	Aug	8	Urban (house)	Hypothermia	11–14 (PMI_min_)	*Calliphora vicina*	Proventriculus, yolk mass in thin gut loops	Thin midgut (at least)
11	Dec	Unknown	Urban (house)	Combined drug toxicity	Unknown	*Chrysomya rufifacies, Ch. varipes*	Ventral nerve cord fully contracted, pigmented cephalopharyngeal skeleton, tracheoles, muscle, thin gut loops, proventriculus	Trachea mouth hooks

A variety of species were reared from the aged egg samples, and these showed the same anatomical features as have been identified from *C. vicina*. Eggs of the calliphorid species *C. stygia* (Fabricius), *Chrysomya varipes* (Macquart) and *Ch. rufifacies* (Macquart) (Diptera: Calliphoridae) were sectioned, in addition to those of *C. vicina*. We were not able to compare the estimated egg stage timing with the minimum post-mortem interval estimated for each case. This was because these case opinions were completed using older life history stages than the eggs.

## Discussion

Histological techniques may appear time-consuming and challenging for those unfamiliar with them, but this is not the case once experience is gained in this methodology. There is real value in using these techniques, especially with help from an experienced histologist, because temporal resolution for aging eggs with morphological analysis is potentially superior to that of other techniques. We developed a technically simple histological method that allows clear visualization of organogenesis, and many other key developmental events within the eggs of *C. vicina*. The method was developed within a busy forensic histology laboratory, and therefore maximizes ease and efficiency. Cost is also likely to be reduced in comparison to molecular techniques [13], especially when processing large numbers of specimens. Moreover, an experienced histologist could produce the required sections rapidly for either research or casework.

The features that we identified correlated with those seen in other histological studies [11,12,17–19,21,28], and the anatomical detail described even in this preliminary study was sufficient to determine age to within 6 h in our blind trial at 20 °C. Attempts to further narrow the age range estimate to within 1–3 h resulted in discrepancies of up to 3 h in one-third of cases, which clearly shows that further research is required before the technique can be used to provide precise answers concerning egg age. This was especially the case in our 15 °C cohort, with some stages coinciding (e.g. migration, blastoderm and pseudo-segmentation all occurred at T_9_). This was due to natural variation in development and because our sampling points were widely spaced due to the preliminary nature of the work. The proportion of eggs in the various stages of development at the same time point also could not be scored as the egg numbers were not controlled. Future studies will tighten the time-frames that we used to more accurately time developmental stages. Also, the ease of employing our technique will certainly allow the mass embedding and sectioning of eggs that is required to create a more detailed timeline.

The structures and developmental processes that we did not see were usually those that were small or subtle. They have therefore been lost between section levels due to their small size, or appeared so fleetingly as to have evaded sampling due to our relatively coarse sampling interval resolution. Plane of section also made some features more obvious, for example the germ band was seen definitively on transverse section only. Further morphological research, and the possible incorporation of special stains, may enable the identification of some of these features in other planes of section. Other small structures may require better cellular detail than we could produce for visualization. This problem may be ameliorated by the use of 5 s hot water fixation prior to storage, which also has the added advantage of guaranteeing instant cessation of embryogenesis. There is some evidence that development may continue for an unknown time when eggs are preserved in ethanol, even if ethylenediaminetetraacetic acid (EDTA) and dimethyl sulfoxide (DMSO) are added (personal comm. Gail S. Anderson and Sherah VanLaerhoven). We saw no obvious evidence of post-preservation development, but acknowledge that our infrequent sampling interval may not have detected this phenomenon. Further experimental evaluation of this potential problem is currently underway, and until the results are available, any contemplation of preserving eggs for histological examination in a forensic case should include the use of 5 s hot water fixation.

Many organs were also seen by Van Der Starre-Van Der Molen [12,23] at earlier stages of development than in the current study. This includes elements of the gastric, respiratory and neural systems. Part of this problem is likely to be the small size of these structures at early stages, but the inferior resolution of cellular structure resulting from our technique is also likely to contribute. The artefacts we encountered here resulted not only in some tissue destruction, but also most likely obscured some subtle details. As mentioned above, the routine use of hot water fixation may assist with this problem. It is also possible that stage-specific special stains could be integrated to further delineate anatomical details if a “screening” H&E section is produced from the first level of section, and used to determine developmental stage before further levels are produced.

The current study was focussed on proof of concept and method development and some of the problems encountered may be resolved with future work. We have established that our technique will be suitable for use in casework, even for samples up to 11 years old. However, evidence collection should be carried out as early as possible since eggs do develop rapidly, especially at warm temperatures. Also, there is evidence of divergence of development stage over time with the same cohort [12], so a more precise age estimate might be obtained with a younger sample. Also, if this technique is to be used for casework we recommend extreme caution at this early stage. But eggs can certainly be preserved now, or even embedded and/or sectioned for potential future analysis when the quality of available data improves.

The percentage of precocious eggs in *C. vicina* has been measured at 2.55% in egg batches from laboratory populations, and 8.82% in egg batches from wild populations in one British study [30]. Our results suggest that it is relatively easy to determine whether a laboratory-produced egg is precocious because it is markedly overdeveloped compared with the eggs around it, which tended to develop within a more narrow spread of stages. However, it is likely that there is a range of degrees of embryological precocity, and we therefore need to quantify its histological range and incidence. Conversely, there were a small number of eggs that appeared to have died during development, or were notably slower than their cohort. This phenomenon was also noted by Van Der Starre-Van Der Molen, in tandem with developmental defects [12], which we did not note. We must therefore quantify this phenomenon in both wild populations and research colonies used to generate eggs.

Future work will also investigate the use of special stains, such as Masson Trichrome (MT) and Periodic Acid Schiff (PAS). Our current choice of stain was based on the fact that the H&E stain is the routine histological stain used in the vast majority of histology laboratories. Special stains may be superior for highlighting certain tissue types and substances. MT has already been used with great success in the flea [35], and PAS has been used by Van Der Starre-Van Der Molen to examine changes in the glycogen distribution within the developing embryo. Many organs require stored glycogen to provide energy for development, and there are a number of organs in the blow fly embryo that appear to rapidly digest their initial starting glycogen store [22]. This may be useful especially in aging young embryos, and it would be feasible in casework to perform PAS or other special stains on serial sections at each level.

Another potentially interesting adjunct to this work could be the use of novel non-invasive and non-destructive imaging methods for the aging of blow fly eggs, such as Micro-Computed Tomography and Optical Coherence Tomography. These could be used to further complement our histological technique. Early studies using these methods have been conducted on blow fly pupae [36–38] and these techniques may also potentially be applicable to the egg stage.

There were some technical problems encountered with the sections that are deemed to be an inevitable trade-off for ease and speed. These are mainly concerned with fixation and processing (e.g. tissue shrinkage due to dehydration), and with sectioning of the relatively hard chorion, which is dissected or soaked off the egg by other authors in order to avoid this problem [10,12]. Common sectioning artefacts were shattering of the sections, knife lines and tissue loss. The first two artefacts are generally caused by cutting on a dulled or damaged blade. Changing to a new sharp blade should prevent these artefacts from occurring, however due to the nature of the tissue, blade replacement will need to occur regularly. Tissue shrinkage (compression) may also be caused by rapid sectioning. Therefore, when sections were cut very slowly and carefully, this greatly minimized this artefact. A recent publication describes a simple method of dechorionating eggs [10], which may also be transferable to histology studies, and may eliminate some sectioning artefacts. Fixation of dechorionated eggs may also be enhanced, however trials are required to test this possibility.

Tissue loss was not a significant problem with this method, although it did occur; only 7% of all slides had tissue loss that prevented the aging of an embryo. There are multiple ways of reducing this problem, and we cut sections slowly, floated them on the water bath for an additional 5–10 s, and oven dried them overnight. However, “Sticky slides” were not used here, and could potentially be tried in future studies. These positively charged slides are coated in (3-aminopropyl) triethoxysilane (commonly called AAS or AES), and may provide increased adherence to the slide. Gelatine slides may also be used as a cheap alternative to sticky slides.
